# Isolation and Structural Characterization of a Novel Antioxidant Mannoglucan from a Marine Bubble Snail, *Bullacta exarata* (Philippi)

**DOI:** 10.3390/md11114464

**Published:** 2013-11-11

**Authors:** Donghong Liu, Ningbo Liao, Xingqian Ye, Yaqin Hu, Dan Wu, Xin Guo, Jianjun Zhong, Jianyong Wu, Shiguo Chen

**Affiliations:** 1 College of Biosystem Engineering and Food Science, Zhejiang University, 866 Yuhangtang Road, Xihu District, Hangzhou 310058, China; E-Mails: dhliu@zju.edu.cn (D.L.); liaoningbo2010@sina.com (N.L.); psu@zju.edu.cn (X.Y.); processing@zju.edu.cn (D.W.); crystalgx0509@gmail.com (X.G.); hendle527@yahoo.com (J.Z.); chenshiguo210@zju.edu.cn (S.C.); 2 Fuli Institute of Food Science, Zhejiang University, Hangzhou 310058, China; 3 Department of Applied Biology & Chemical Technology, The Hong Kong Polytechnic University, Hung Hom 990077, Kowloon, Hong Kong; E-Mail: jian-yong.wu@polyu.edu.hk

**Keywords:** *Bullacta exarata*, structure, polysaccharide, mannoglucan

## Abstract

*Bullacta exarata* is one of the most economically important aquatic species in China, noted for not only its delicious taste and nutritional value, but also for its pharmacological activities. In order to explore its potential in medical applications, a mannoglucan designated as BEPS-IB was isolated and purified from the foot muscle of *B. exarata* after papain digestion. Chemical composition analysis indicated BEPS-IB contained mainly d-glucose and d-mannose in a molar ratio of 1:0.52, with an average molecular weight of about 94 kDa. The linkage information was determined by methylation analysis, and the anomeric configuration and chain linkage were confirmed by IR and 2D NMR. The results indicated BEPS-IB was composed of Glc*p*_6_Man*p* heptasaccharide repeating unit in the backbone, with occasional branch chains of mannose residues (14%) occurring in the backbone mannose. Further antioxidant assay indicated BEPS-IB exhibited positive antioxidant activity in scavenging superoxide radicals and reducing power. This is the first report on the structure and bioactivity of the mannoglucan from the *B. exarata*.

## 1. Introduction

There has been an increasing interest in recent years in the structures and functions of natural polysaccharides from various sources because of their nutraceutical and pharmaceutical potential. Glucans, a kind of neutral polysaccharides mainly consisting of glucose monomers, have been widely isolated from medical herb or fungus. They have been reported to stimulate the immune system and decrease infectious complications in humans [1,2,3] and experimental animals [4]. These activities were related to their structural features. For example, β-(1→6)-linked side chains of glucose residues increases antitumor activity [5], and a β-(1→3)-linked backbone seems essential for the antioxidant effect [6,7]. However, most of the reports on the glucans were focused on medical herb or fungi sources, with little knowledge on the structure and function of the glucans in the animals, especially the marine invertebrates.

Polysaccharides from mollusks have attracted increasing attention in recent years because of their potential pharmaceutical values, such as anti-tumor [8], immunity-enhancement [9], anti-inflammation [10] and anti-aging effects [11]. Several bioactive glucans from the mollusks have also been reported, e.g., Zhang, Ye and Wang [12] reported a water soluble α-glucan from the soft body of *Bellamya purificata* with a main chain of (1→4)-linked d-glucopyranosyl and branching points at O-6 of (1→6)-linked d-glucopyranosyl residues, which showed significant anti-inflammatory activity. A glucan from *Cyclina sinensis* also showed antioxidant and hepatoprotective activities [13].

*Bullacta exarata* (Philippi), generally called Tutie or Niluo in China, belongs to the Mollusca phylum, Gastropoda class, and Haminoeidae family, which is a species of bubble snail with a bullate, spirally striate shell found in the coastlines of the South and East China Seas. As an important economic resource in eastern China, *B. exarata* is noted not only for its delicious taste and nutritional value, but also for its pharmacological activities. It is a highly acclaimed species in traditional Chinese medicine (TCM) with a broad spectrum of health promoting effects on the kidney, lung, liver and immune functions [14,15,16]. The foot muscle is the main edible part of *B. exarata*, and its abundance in proteins and carbohydrates makes it attractive for exploiting. However, the structure and bioactivities of the polysaccharides from this species are rarely researched and little is known about them.

In the present study, a mannoglucan was isolated from *B. exarata* and purified by anion-exchange and gel-filtration chromatography. The sequence of the purified polysaccharide was determined by a combination of composition analysis, methylation analysis, IR and NMR, and its antioxidant activity was investigated by the reducing power assay and the superoxide radical scavenging assay.

## 2. Results and Discussion

### 2.1. Isolation and Purification of Polysaccharides

**Figure 1:**
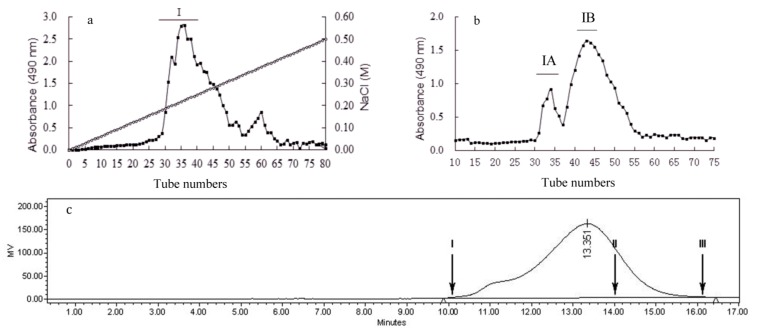
Isolation of the polysaccharides present in the aqueous extract of *B. exarata*. The crude extract was fractionated by ion-exchange chromatography on a DEAE ion-exchange column (**a**) and the collected fraction was further purified by gel filtration chromatography on a Sephacryl S-300 HR column (**b**). Solid bars indicate the fractions collected. The molecular weight of polysaccharide fraction BEPS-IB (Mw = 94 kDa) was determined by HPLC on a TSK-Gel G4000 PWXL column, eluted with 0.2 mol/L NaCl at 0.5 mL/min (**c**). Range of molecular weight in kDa: I = 500; II = 66.9; III = 40.

The extraction of *B. exarata* foot muscle by papain digestion retained an extract yield of 7.3% (w/w) by dry weight, which was named as CBEPS. It was further fractionated by ion exchange chromatography on a DEAE-52 column (Figure 1a). The major peak labeled as BEPS-I (3.62%, w/w) was collected and further purified on a Sephacryl S-300 HR gel-permeation chromatography, two factions designated as BEPS-IA (1.03%, w/w) and BEPS-IB (2.12%, w/w) was collected (Figure 1b). The main fraction BEPS-IB showed a single peak on the GPC (Figure 1c), corresponding to an average molecular weight around 94 kDa.

Chemical composition analysis indicated the BEPS-IB showed a total carbohydrate of 98.7% by the phenol-sulfuric acid method, indicating the removing of the protein part after papain digestion. Further monosaccharide composition analysis by the HPLC-PMP method indicated BEPS-IB had a simpler composition than the crude polysaccharides after extensive purification, which was mainly composed of Glc and Man, with a proportion of 1:0.52. A small proportion of Gal and Fuc was also detected (Table 1). The protein content of BEPS-IB decreased from 10.7 to 2.1 after purification, whereas the sulfated was around 0.47%–1.5% (w/w) in all the tested fractions.

**Table 1:** Yields, protein contents, sugar contents, sulfate contents and Mw of *B. exarata* polysaccharides.

Composition	Samples			
	^a^ CBEPS	BEPS-I	BEPS-IA	BEPS-IB
Yield (%)	7.3	3.6	1.03	2.2
Neutral sugar (%)	80.12	89.31	76.29	98.76
Protein (%)	10.7	2.4	2.7	2.1
Sulfate (%)	1.2	1.5	0.47	1.23
Mw (kDa)	-- ^b^	-- ^b^	127	94
Molar ratio of monosaccharides				
Mannose	0.74	1.69	0.32	0.52
Glucose	0.43	1.26	0.57	1
Galactose	0.32	0.03	0.04	0.03
Fucose	0.18	0.12	ND	0.01
Rhamnose	0.56	0.22	0.31	ND
Arabinose	0.17	0.07	0.03	ND

^a^ Crude *B. exarata* polysaccharide; ^b^ The polysaccharide was a mixture; ND, not detected.

#### 2.1.1. IR Spectrum and Elucidation of BEPS-IB

**Figure 2:**
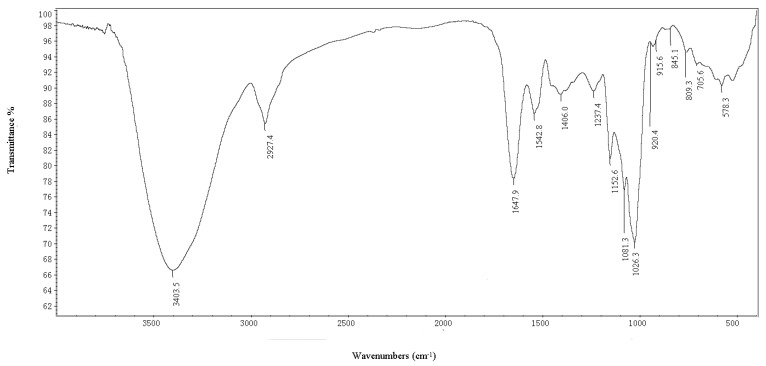
Infrared spectra of polysaccharide (BEPS-IB) from *B. exarata.*

Figure 2 presents the IR spectrum of BEPS-IB. The broad and intense stretching at 3400 cm^−1^ is characteristic of hydroxyl groups, and the weak stretching at 2930 cm^−1^ is attributed to the C–H bond [17]. The band at 1647 cm^−1^ can be attributed to water bound to the polysaccharide molecule, and the bands between 950 and 1200 cm^−1^ are mostly attributed to C–O–C and C–O–H linkages [18]. Absorptions at 916 cm^−1^ are typical for d-Glc in the pyranose form. The fraction also exhibited an obvious characteristic absorption at 920 and 809 cm^−1^ corresponding to the existence of mannose [19]. Moreover, the characteristic absorptions at 845 cm^−1^ in the IR spectra indicated the presence of α-glycosidic linkages.

#### 2.1.2. Methylation Analysis

Methylation analysis GC-MS suggested BEPS-IB contained terminal Glc, 6-linked Glc, 3-linked Man and 3,6-linked Man (assigned as X, V, U and W) at a molar ratio of 1.32:6.43:3.16:0.97 (Table 2). The presence of the 3,6-linked Man linkages in the polysaccharide chain was indicative of the substitution of the Man residues.

#### 2.1.3. Structure Characteristics of BEPS-IB from NMR

Based on the methylation analysis, the structure characteristics of BEPS-IB, especially the chain linkage and conformation of the sugar units, were completed and confirmed by 1D and 2D NMR.

**Figure 3:**
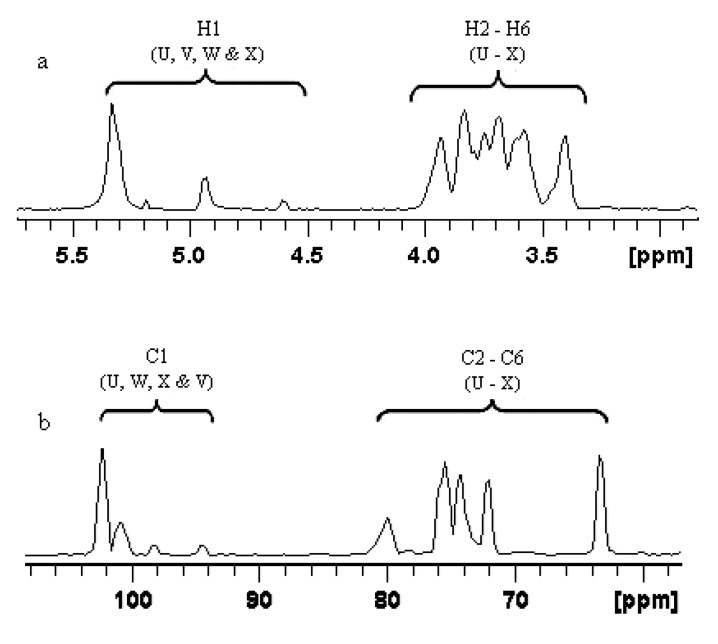
^1^H NMR (**a**) and ^13^C NMR (**b**) spectrum (600 MHz, D_2_O, 60 °C) of BEPS-IB isolated from *B. exarata*.

The ^1^H NMR spectrum (600 MHz) (Figure 3a) of BEPS-IB was recorded at 60 °C. It showed four anomeric proton signals at δ 5.338, 5.192, 4.943 and 4.603 ppm in a molar ratio of about 6:1:3:1 (Figure 3a), which were assigned to the four types of sugar units obtained in the methylation analysis (U, V, W and X). The signals around 3.5–4.5 ppm were assigned to cross ring protons (Figure 3a). Similarly, the ^13^C spectrum (Figure 3b) showed four signals δ 102.2, 100.8, 98.2 and 94.6 ppm in a molar ratio of nearly 6:1:1:3, which were assigned to the anomeric carbon of sugar units U, V, W and V, respectively. The other signals around 60–85 ppm were also assigned to the carbon signals for C2–C5 (Figure 3b).

**Table 2:** GC-MS data for alditol acetate derivatives from methylated polysaccharide (BEPS-IB) isolated from *B. exarata*.

Methylated sugar	Retention	Molar ratio	Mass fragment (*m*/*z*)	Type of linkage
	time (min)			
2,3,4,6-tetra-*O*-Me-Glc ^a^	14.80	1.32	43, 45, 71, 87, 101, 117, 129, 145, 161, 205	Glc-(1→
2,4,6-tri-*O*-Me-Man	16.95	3.16	43, 45, 87, 101, 117, 129, 161, 233	→3)-Man-(1→
2,3,4-tri-*O*-Me-Glc	17.31	6.43	43, 45, 71, 87, 101, 117, 129, 161, 173, 189, 233	→6)-Glc-(1→
2,4-tri-*O*-Me-Man	19.16	0.97	43, 87, 101, 117, 129, 189	→3,6)-Man-(1→

^a^ 2,3,4,6-tetra-*O*-Me-Glc = 1,5-di-*O*-acetyl-2,3,4,6-tetra-*O*-methyl-glucose.

**Figure 4:**
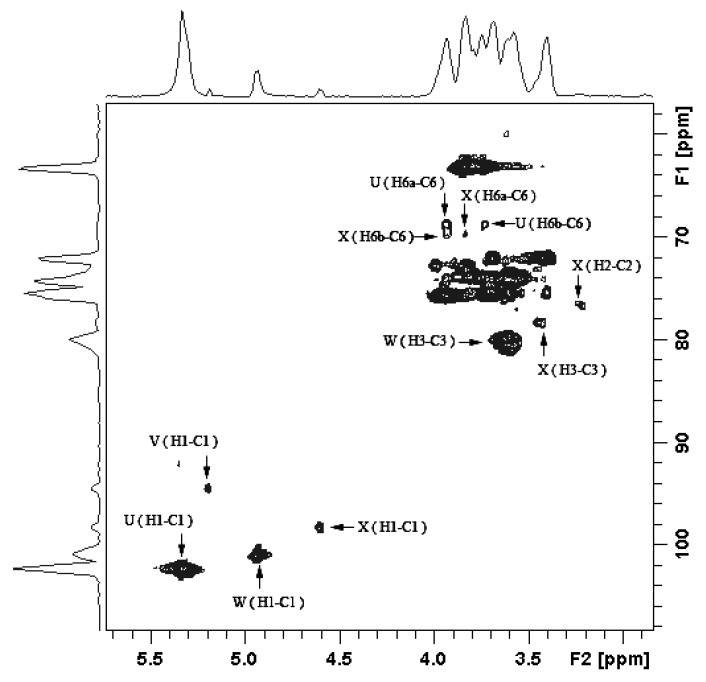
HMQC spectrum of BEPS-IB isolated from *B. exarata*.

The complete assignment of the chemicals-shifts of the sugar units were obtained from 2D NMR (Table 3), including ^1^H-^1^H COSY (Supplementary Figure S1), TOCSY (Supplementary Figure S2a), NOESY (Supplementary Figure S2b), ^1^H-^13^C HMBC (data not shown) and HMQC (Figure 4), according to the published methodology [20]. The down-shifts in carbon signals of the sugar units compared to the native glucan may suggest possible linkage information, e.g., the down-shift in the C-3 position of the unit W indicated it may be 3-linked Man. Similarly, unit U, V and X were deduced as 6-linked Glc, terminal Glc and 3,6-linked Man, respectively.

**Table 3:** The ^1^H and ^13^C NMR chemical shifts for the polysaccharide BEPS-IB isolated from *B. exarata*.

Glycosidic linkage		Chemical shifts (ppm)					
		H-1 (C-1)	H-2 (C-2)	H-3 (C-3)	H-4 (C-4)	H-5 (C-5)	H-6 (C-6)
U	→6)-α-Glc*p*-(1→	5.338 (102.2)	3.587 (72.1)	3.742 (76.8)	3.632 (73.2)	3.964 (76.8)	3.937/3.736 (68.9)
V	α-Glc*p*-(1→	5.192 (94.6)	3.517 (73.8)	3.811 (72.4)	3.342 (73.2)	3.623 (75.3)	3.831/3.792 (63.2)
W	→3)-α-Man-(1→	4.943 (100.8)	3.542 (75.4)	3.605 (80.1)	3.614 (74.8)	3.963 (76.3)	3.972/3.752 (69.2)
X	→3,6)-α-Man-(1→	4.603 (98.2)	3.211 (76.5)	3.729 (78.5)	3.412 (73.9)	3.574 (76.7)	3.833/3.925 (69.6)

The linkage information of the sugar units were further confirmed by NOESY and HMBC experiments. In the HMBC spectrum, the intra- and inter-residual connectivities of both anomeric protons and carbons of each of the glycosyl residues were summarized in Table 4. Cross peaks indicated the correlation signals were found between C-1 of residue U (δ 102.2) with H-6 of residue X (U C-1, X H-6), H-1 of residue U (δ 5.338) with C-6 of residue X (U H-1, X C-6), indicating the U was linked to the 6-position of the X. Similarly, the correlation signals W H-1–X C-3 indicated the unit W linked to the 3-position of X. Thus, both 3 and 6 positions of X were substituted. The correlation signals V H-1–W C-3 and V C-1–W H-3 indicated unit V linked to the 3-position of the unit W; an intraresidual coupling between H-1 of residue U with its own C-6 (U H-1, U C-6) indicated that the 6-linkage was among different U repeats. In the NOESY spectrum (Supplementary Figure S2b), similar correlations of the protons were observed: U1–X6, V1–W3, W1–X3 and X1–U6, which confirmed the above results from HMBC.

**Table 4:** The connectivities observed in an HMBC spectrum for the anomeric protons/carbons of the sugar residues of BEPS-IB from *B. exarata*.

Residue	Sugar linkage		Anomeric atom (δ_H_/δ_C_)	Observed connectivities		
				δ_H_/δ_C_	Residue	Atom
U	→6)-α-Glc*p*-(1→		5.338	69.6	U: H-1	X: C-6
				73.2	U: H-1	U: C-4
			102.3	3.83	U: C-1	X: H-6a
				3.63	U: C-1	U: H-4
V	α-Glc*p*-(1→		5.192	80.1	V: H-1	W: C-3
				73.8	V: H-1	V: C-2
				72.4	V: H-1	V: C-3
			94.6	3.61	V: C-1	W: H-3
				3.52	V: C-1	V: H-2
				3.81	V: C-1	V: H-3
W	→3)-α-Man-(1→		4.943	78.5	W: H-1	X: C-3
				76.3	W: H-1	W: C-5
			100.8	3.96	W: C-1	W: H-5
X	→3,6)-α-Man-(1→		4.603	68.9	X: H-1	U: C-6
				3.94	X: C-1	U: H-6a
			98.2	3.21	X: C-2	X: H-2

**Figure 5:**
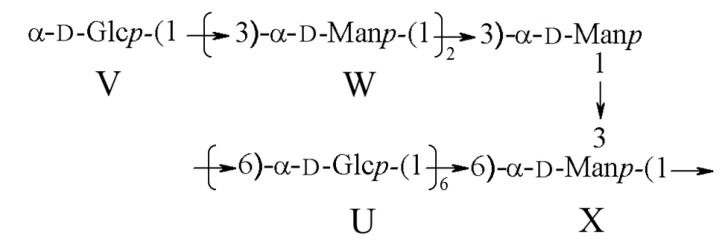
Proposed structural features of the BEPS-IB isolated from *B. exarata*.

Based on all these results from methylation analysis and 2D NMR, the main repeating unit structure of BEPS-IB was deduced and is shown in Figure 5.

### 2.2. Antioxidant Activity

**Figure 6:**
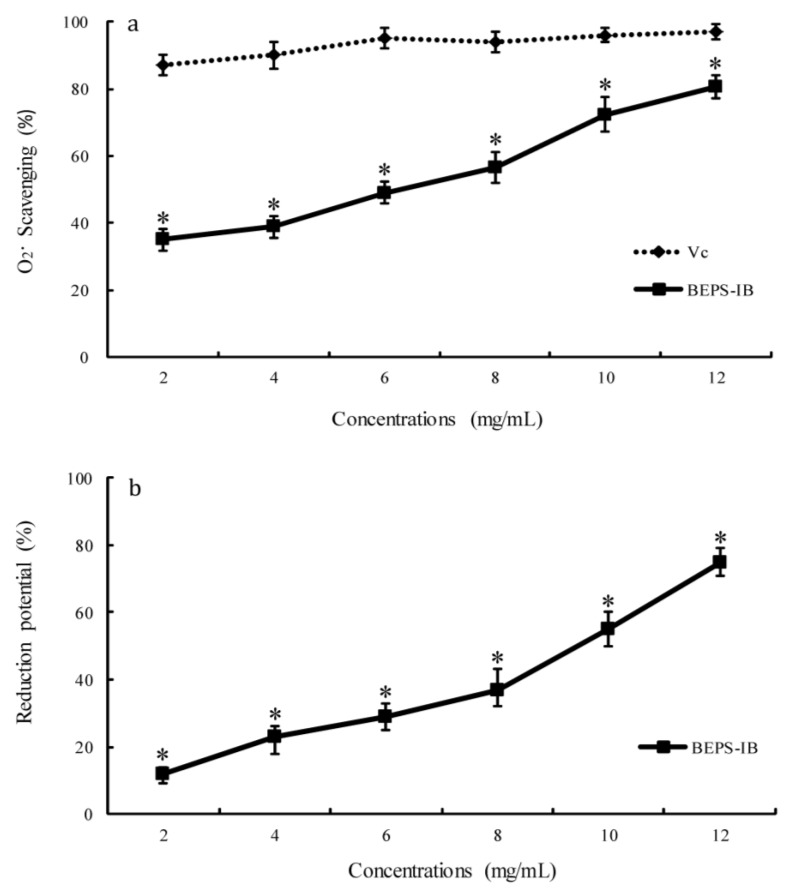
Antioxidant activity of the BEPS-IB. (**a**) Scavenging effects of BEPS-IB on superoxide radical (O_2_^•^); (**b**) Reducing power. Values are means ± SD (*n* = 3). Significant differences from the control were evaluated using Student’s *t*-test: * *p* < 0.05. Reducing power was expressed as a percentage of the activity shown by vitamin C.

Figure 6 showed the antioxidant activity of BEPS-IB determined by scavenge superoxide radical assay and reducing power assay. As shown in Figure 6a, BEPS-IB can scavenge superoxide radicals at concentrations between 2 and 12 mg/mL in a dose dependent way. The IC_50_ was 6.23 mg/mL, similar to the glucans isolated from other mollusks, which were usually around 4–10 mg/mL [13]. However, the IC_50_ was much lower than those from medical fungus, which were usually bound with pigments and caused an increase in the antioxidant activity.

In the reducing power assay (Figure 6b), reducing capacity was expressed as a percentage of the activity shown by vitamin C. The reducing capacity was positively correlated with sample concentration. At a concentration of 12 mg/mL, the reducing capacity of BEPS-IB was 75%. The reducing properties are generally associated with the presence of reductones, which have been shown to exert antioxidant action by breaking the free radical chain by donating a hydrogen atom. Our data of the reduction potential suggested that there might be a direct correlation between antioxidant activity and reducing capacity in BEPS-IB.

Reactive oxygen species (ROS), capable of causing damage to DNA, have been associated with carcinogenesis, coronary heart disease, and many other health problems related to advancing age [21,22]. The antioxidant activities of polysaccharides were not a function of a single factor but a combination of several factors, such as content of sulfuric radicals, molecular weight, protein content and type of sugar. The relatively lower potential of the BEPS-IB to other glucans may attribute to the high content of mannose. However, it has been reported that addition of mannose in glucan can also improve the antitumor action of the polysaccharides, as a polysaccharide receptor has been found on human macrophages, which demonstrated high specificity for mannose [23]. Thus, the antitumor potential of polysaccharides needs to be further investigated.

## 3. Materials and Methods

### 3.1. Materials and Reagents

*Bullacta exarata* snails were supplied by Huzhou Lurong Seafood Co., Ltd., China and stored at −20 °C before use. TSK G4000PWXL columns were sourced from TOSOH BIOSEP (Tokyo, Japan), and Sephacryl S-300 HR from Amersham Biosciences (Uppsala, Sweden). Diethyaminoethyl ion-exchange gel was from Whatman (Brentford, UK). Monosaccharides standards and disaccharide lactose were purchased from Sigma (St. Louis, MO, USA). Papain and cystein (Cys) were purchased from Fluka (Seelze, Germany). The derivatization reagent 1-phenyl-3-methyl-5-pyrazolone (PMP) was from Sinopharm Chemical Reagent (Shanghai, China). All other reagents used were analytical grade.

### 3.2. Isolation and Purification of Polysaccharides

The procedure used for the isolation of polysaccharides was similar to previously described [24]. *B. exarata* snails (30 kg) were shelled and the foot muscle was homogenized, and treated with acetone to remove fats (1:1). After centrifugation (6000 rpm, 20 min) and overnight drying, the resulting pellets were kept in distilled water at 60 °C for 8 h with constant stirring. The process was repeated three times. The supernatant was concentrated and precipitated in 4 volumes of ethanol. The precipitate was collected by centrifugation (6000 rpm, 20 min) and dissolved in distilled water and protein was removed by the Sevag method [25]. Then, the crude polysaccharide fraction was obtained by precipitation in 4 volumes of ethanol and washed with acetone and ethyl ether several times. The crude polysaccharide preparation was separated using a DEAE ion-exchange column (2.6 cm × 30 cm), followed with a Sephacryl S-300 gel filtration column (1.6 cm × 100 cm). Carbohydrate content was determined by the phenol/sulfuric acid assay. The isolation procedures were repeated three times and showed no significant influenceon the composition of the polysaccharides.

### 3.3. Chemical Analysis of Polysaccharide Fractions

Estimation of average molecular weights was performed on HPLC using TSK-G4000 and -G3000 PWXL columns, at a sample injection volume of 20 μL (1 mg/mL) and flow rate of 0.5 mL/min on a Waters 2870 system (Milford, MA, USA), with a 2414 refractive index detector. The mobile phase consisted of 0.2 M NaCl. The column was maintained at 40 °C. Gel permeation chromatography (GPC) were recorded on a computer with liquid chromatography (LC) solution version 1.25 software, preliminary calibration of the column was performed using dextrin in a range of molecular weights measured in kDa (I = 500; II = 66.9; III = 48; IV = 20; V = 5; Showa-Denko, Tokyo, Japan). The Breeze™ 2 software was utilized for data acquisition and analysis.

The monosaccharide composition was determined using a PMP-HPLC method [26]. HPLC analyses were performed on an Agilent ZORBAX Eclipse XDB-C18 column (5 μm, 4.6 mm × 150 mm) at 25 °C and UV detection at 250 nm. The mobile phase was 0.05 M KH_2_PO_4_ (pH 6.9) with 15% (solvent A) and 40% (solvent B) acetonitrile in water. A gradient of B from 8% to 19% in 25 min was used. Protein concentration was determined by the Lowry method [27].

### 3.4. Methylation Analysis

BEPS-IB (10 mg) was methylated with the method reported by Needs and Selvendran [28]. The partially methylated sample hydrolyzed by 4 M trifluoroacetic acid at 100 °C for 4 h. The resultant aldoses were reduced to their corresponding alditols by sodium borodeuteride (NaBD_4_). The partially methylated alditols were then acetylated with a pyridine:acetic anhydride (1:1) solution at 100 °C for 1 h. The alditol acetates were analyzed by GC-MS, and the methylated sugar linkages were identified by the retention time and fragmentation pattern [28,29].

### 3.5. Measurement of IR and NMR Spectra

Purified BEPS-IB was deuterium-exchanged by freeze-drying three times and then dissolved in D_2_O to a final concentration of 60 mg/mL. ^1^H NMR spectra of BEPS-IB were measured at 600 MHz, in D_2_O on a Bruker AVANCE III 600 spectrometer at 60 °C, the ^13^C NMR was recorded at room temperature. Signals at δ_H_ 2.22 and δ_C_ 31.1 for acetone were used as external standards. The ^1^H-^1^H and ^1^H-^13^C connectivities were established by two-dimensional NMR (COSY, HMQC, NOESY, HMBC, and TOCSY).

An infrared spectrum of the polysaccharide (2 mg) was recorded on a Perkin-Elmer instrument in KBr pellets at room temperature.

### 3.6. Antioxidant Activity Assays

The antioxidant activity of BEPS-IB was tested with two *in vitro* assays: The reducing power assay and the superoxide radical scavenging assay. The reducing power assay was performed as described by Li, Zhou and Li [30] with modifications. Briefly, 1 mL samples of different concentrations (2–12 mg/mL) in phosphate buffer (0.2 M, pH 6.6) were mixed with 1 mL potassium ferricyanide (1%, w/v), and incubated at 50 °C for 20 min. The reaction was terminated by the addition of 1 mL trichloroacetic acid (10%, w/v) to the mixture and the solution was mixed with 0.2 mL ferric chloride (0.1%, w/v) and the absorbance was measured at 700 nm. Reducing power was expressed as a percentage of the activity shown by a 1 mM solution of vitamin C.

The superoxide radical scavenging activity assay was performed using the method of photoreduction of NBT (nitroblue tetrazolium) [31], with some modifications. Superoxide radicals were generated in 3 mL phosphate buffer (0.1 M, pH 7.4) containing 156 μM nicotinamide adenine dinucleotide (NADH) (reduced form), 52 μM nitrotetrazolium blue chloride (NBT), 20 μM phenazin methosulfate, and varying concentrations of polysaccharides (2–12 mg/mL). The color reaction of superoxide radicals and NBT was detected by monitoring the absorbance at 560 nm. Vitamin C was used as reference material. In the essential control, NADH was substituted with phosphate buffer. The inhibition percentage was calculated using the following formula:

Scavenging effect (%) = (1 − A_Sample 560_/A_Control 560_) × 100

### 3.7. Statistical Analysis

The data were reported as mean ± standard deviation (SD) (*n* = 3) and evaluated by one-way analysis of variance (ANOVA) followed by the Student’s *t*-test. Differences were considered to be statistically significant if *p* < 0.05. All statistical analyses were carried out using Statistical Product and Service Solutions (SPSS) for Windows, Version 16.0 (SPSS Inc., Chicago, IL, USA).

## 4. Conclusions

A novel mannoglucan designated as BEPS-IB has been isolated and purified from the foot muscle of *B. exarata*. Chemical composition analysis indicated that BEPS-IB mainly consisted of glucose, mannose and minor contents of galactose and fucose, with a molar ratio of 1:0.52:0.03:0.01. The complete molecular structure was established through several experiments, including methylation analysis, NMR and IR spectra. The results indicated that BEPS-IB was a heptasaccharide backbone ([–(1→6Glcα)_6_1→6Manα–]_n_), with a tetrasaccharides branch ([–(1→3Manα)_3_1→3Glcα–]*_n_*) occurring at the O-3 position of mannose residues in the backbone. The purified polysaccharide also showed significant antioxidant activity in scavenging superoxide radicals (O_2_^•^) and reducing power. The present study has demonstrated the potential of *B. exarata* as a rich and promising source of novel bioactive polysaccharides.

## Abbreviations

BEPS: crude polysaccharide extracted from *Bullacta exarata*
BEPS-IB: purified *B. exarata* polysaccharide of molecular weight 94 kDa
Fuc: fucose
Gal: galactose
Man: mannose
Glc: glucose
Arb: arabinose
Rha: rhamnose
Xyl: xylose
GalN: galactosamine
GlcN: glucosamine
GlcA: glucuronic acid
GalA: galacturonic acid

## Supplementary Files

Supplementary File 1 Supplementary Materials (PDF, 152 KB)
