# Supplementary material for: Isolation and Structural Characterization of a Novel Antioxidant Mannoglucan from a Marine Bubble Snail, Bullacta exarata (Philippi)
Source: Mar Drugs. 2013 Nov 11;11(11):4464–77. doi: 10.3390/md11114464 (PMC3853739; doi:10.3390/md11114464)

Supplementary Materials

Figure S1. H/H-COSY spectra of BEPS-IB.

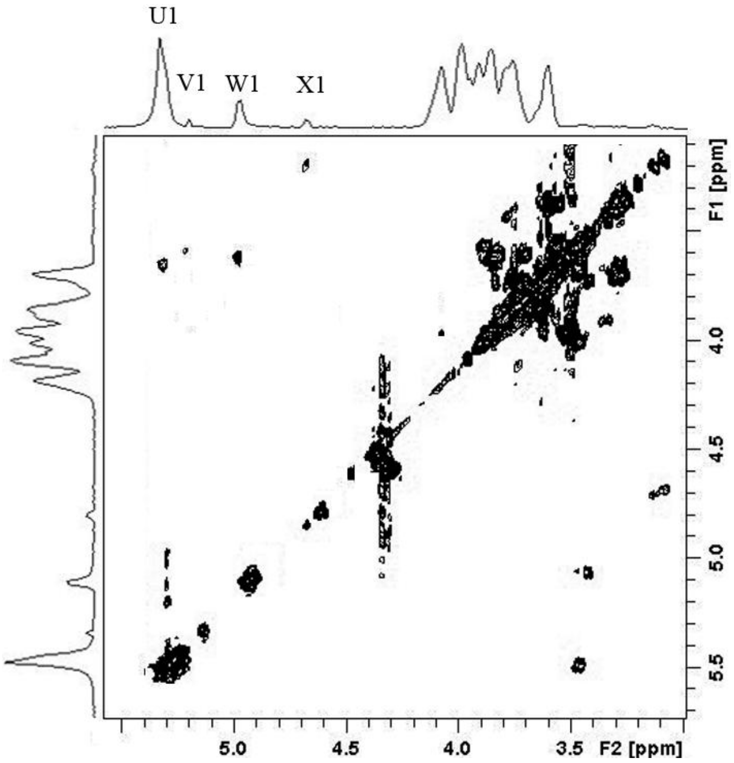

Figure S2. H/H-TOCSY (**a**) and NOESY (**b**) spectra of BEPS-IB.

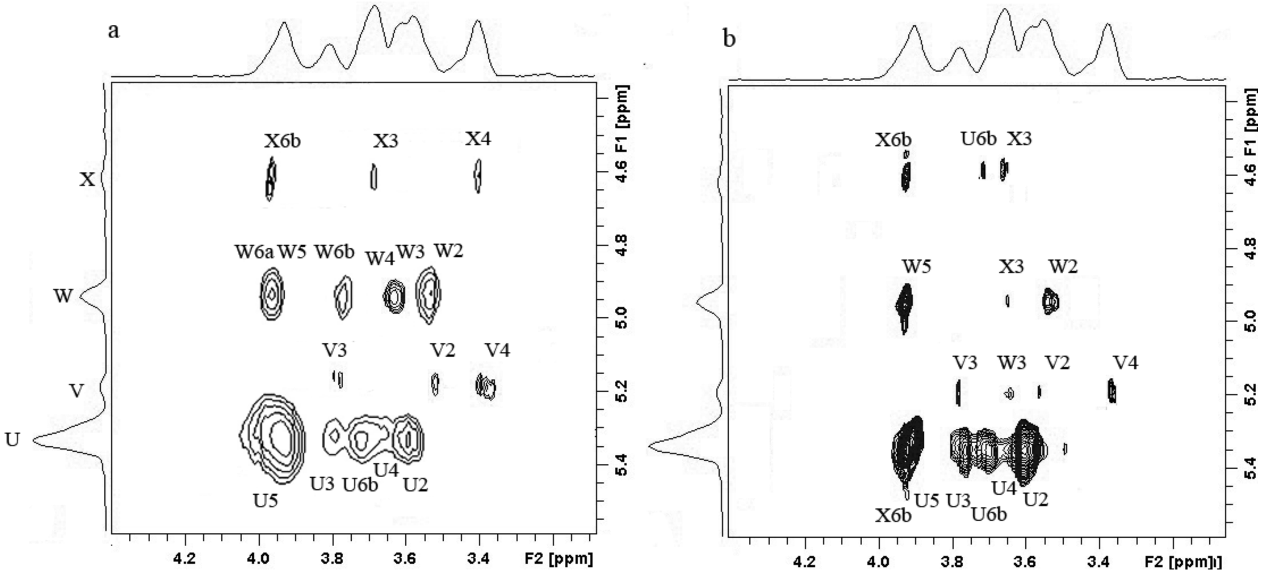

Supplement: Supplementary File 1 — Supplementary Materials (PDF, 152 KB) [file marinedrugs-11-04464-s001.pdf]
